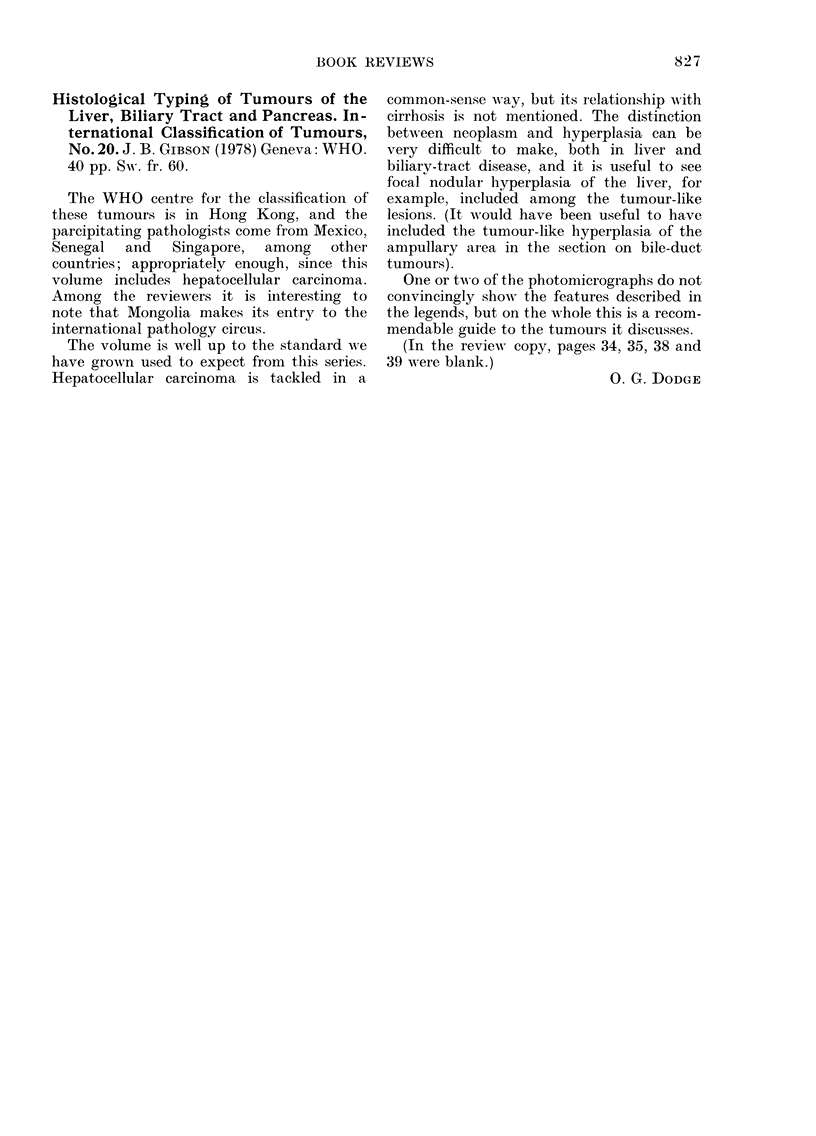# Histological Typing of Tumours of the Liver, Biliary Tract and Pancreas. International Classification of Tumours, No. 20

**Published:** 1979-11

**Authors:** O. G. Dodge


**Histological Typing of Tumours of the Liver, Biliary Tract and Pancreas. International Classification of Tumours, No. 20.** J. B. Gibson (1978) Geneva: WHO. 40 pp. Sw. fr. 60.

The WHO centre for the classification of these tumours is in Hong Kong, and the parcipitating pathologists come from Mexico, Senegal and Singapore, among other countries; appropriately enough, since this volume includes hepatocellular carcinoma. Among the reviewers it is interesting to note that Mongolia makes its entry to the international pathology circus.

The volume is well up to the standard we have grown used to expect from this series. Hepatocellular carcinoma is tackled in a common-sense way, but its relationship with cirrhosis is not mentioned. The distinction between neoplasm and hyperplasia can be very difficult to make, both in liver and biliary-tract disease, and it is useful to see focal nodular hyperplasia of the liver, for example, included among the tumour-like lesions. (It would have been useful to have included the tumour-like hyperplasia of the ampullary area in the section on bile-duct tumours).

One or two of the photomicrographs do not convincingly show the features described in the legends, but on the whole this is a recommendable guide to the tumours it discusses.

(In the review copy, pages 34, 35, 38 and 39 were blank.)

O. G. Dodge